# Deciphering the Virome of *Culex vishnui* Subgroup Mosquitoes, the Major Vectors of Japanese Encephalitis, in Japan

**DOI:** 10.3390/v12030264

**Published:** 2020-02-28

**Authors:** Astri Nur Faizah, Daisuke Kobayashi, Haruhiko Isawa, Michael Amoa-Bosompem, Katsunori Murota, Yukiko Higa, Kyoko Futami, Satoshi Shimada, Kyeong Soon Kim, Kentaro Itokawa, Mamoru Watanabe, Yoshio Tsuda, Noboru Minakawa, Kozue Miura, Kazuhiro Hirayama, Kyoko Sawabe

**Affiliations:** 1 Laboratory of Veterinary Public Health, Graduate School of Agricultural and Life Sciences, The University of Tokyo, 1-1-1 Yayoi, Bunkyo-ku, Tokyo 113-8657, Japan; astrinf@nih.go.jp (A.N.F.); akozue@mail.ecc.u-tokyo.ac.jp (K.M.); 2 Department of Medical Entomology, National Institute of Infectious Diseases, 1-23-1 Toyama, Shinjuku-ku, Tokyo 162-8640, Japan; dkoba@nih.go.jp (D.K.); mamoabosompem@gmail.com (M.A.-B.); k.murota@affrc.go.jp (K.M.); saperoi@nih.go.jp (Y.H.); tabanus-wata@titan.ocn.ne.jp (M.W.); tsuda.yoshio@lecinc.co.jp (Y.T.); sawabe@nih.go.jp (K.S.); 3 Department of Research Promotion, Japan Agency for Medical Research and Development, 20F Yomiuri Shimbun Bldg. 1-7-1 Otemachi, Chiyoda-ku, Tokyo 100-0004, Japan; 4 Department of Environmental Parasitology, Tokyo Medical and Dental University, 1-5-45 Yushima, Bunkyo-ku, Tokyo 113-8510, Japan; 5 Kyushu Research Station, National Institute of Animal Health, NARO, 2702 Chuzan, Kagoshima 891-0105, Japan; 6 Department of Vector Ecology and Environment, Institute of Tropical Medicine, Nagasaki University, 1-12-4 Sakamoto, Nagasaki 852-8523, Japan; futami@nagasaki-u.ac.jp (K.F.); Minakawa@nagasaki-u.ac.jp (N.M.); 7 Department of Virology, Institute of Tropical Medicine, Nagasaki University, 1-12-4 Sakamoto, Nagasaki 852-8523, Japan; kanomkrok77@gmail.com; 8 Joint Department of Veterinary Medicine, Faculty of Agriculture, Tottori University, 4-101 Koyama-cho Minami, Tottori 680-8550, Japan; kim@tottori-u.ac.jp; 9 Pathogen Genomics Center, National Institute of Infectious Diseases, 1-23-1 Toyama, Shinjuku-ku, Tokyo 162-8640, Japan; itokawa@nih.go.jp

**Keywords:** virome, *Culex tritaeniorhynchus*, Japanese encephalitis virus, flavivirus, metagenomics, insect-specific virus, arbovirus, *Culex vishnui* subgroup

## Abstract

Japanese encephalitis (JE) remains a public health concern in several countries, and the *Culex* mosquito plays a central role in its transmission cycle. *Culex* mosquitoes harbor a wide range of viruses, including insect-specific viruses (ISVs), and can transmit a variety of arthropod-borne viruses (arboviruses) that cause human and animal diseases. The current trend of studies displays enhanced efforts to characterize the mosquito virome through bulk RNA sequencing due to possible arbovirus–ISV interactions; however, the extent of viral diversity in the mosquito taxon is still poorly understood, particularly in some disease vectors. In this study, arboviral screening and RNA virome analysis of *Culex tritaeniorhynchus* and *C*. *pseudovishnui*, which are part of the *Culex vishnui* subgroup mosquitoes, were performed. Results from these two mosquito species, known as the major vectors of JE virus (JEV) in Asia, collected in three prefectures in Japan were also compared with the sympatric species *C*. *inatomii*. A total of 27 viruses, including JEV, were detected from these *Culex* mosquitoes. Molecular and phylogenetic analyses of the detected viruses classified 15 of the 27 viruses as novel species, notably belonging to the *Flaviviridae*, *Rhabdoviridae*, *Totiviridae*, and *Iflaviridae* families. The successful isolation of JEV genotype I confirmed its continuous presence in Japan, suggesting the need for periodic surveillance. Aside from JEV, this study has also reported the diversity of the RNA virome of disease vectors and broadened the knowledge on mosquito virome profiles containing both arbovirus and ISV. Mosquito taxon seemed to contribute largely to the virome structure (e.g., virome composition, diversity, and abundance) as opposed to the geographical location of the mosquito species. This study therefore offers notable insights into the ecology and evolution of each identified virus and viral family. To the authors’ knowledge, this is the first study to characterize the viromes of the major JE vectors in Japan.

## 1. Introduction

Approximately 67,900 Japanese encephalitis (JE) cases typically occur annually in 24 JE-endemic countries, with an overall incidence of 1.8 per 100,000 [1]. As the name implies, it has historically been associated with Japan, as the first clinical case and isolation of JE were both recorded in Japan in 1871 and 1935, respectively [2,3]. The disease is caused by infection with JE virus (JEV), a mosquito-borne flavivirus, which is mainly transmitted by mosquitoes of the *Culex vishnui* subgroup [4,5]. The role of *Culex tritaeniorhynchus*, a member of the *C. vishnui* subgroup, as the most important JE vector was first reported in 1938 [6]. *Culex pseudovishnui*, another species in the *C. vishnui* subgroup, was later found to be involved in the transmission [4] and is currently regarded as a major vector of JE.

The application of next-generation sequencing (NGS) technology has enabled broad surveys of viral diversity [7] and thus has significantly sped up mosquito virome analysis in addition to disease surveillance. Although most of the detected viruses are considered insect specific and have no direct impact on public health [8], some of them may have the potential to serve as “natural competitors” to pathogens within mosquitoes. It has been hypothesized that the effects of these insect-specific viruses (ISVs), which can be used as biocontrols, include superinfection exclusion, antiviral immune response enhancement, and vertical transmission in mosquitoes [9]. Furthermore, a number of studies have reported the potential ability of ISV to modulate the transmission of viruses that are pathogenic to vertebrates by reducing the competence of the vector in transmitting arboviruses or modifying the host immune response to subsequent arbovirus infection [10,11,12]. As more evidence showed that the microbiome of the mosquito can alter the susceptibility to certain arboviruses [13], mosquito virome studies have significantly risen.

Emerging efforts for mosquito virome determination and characterization have been recorded in some parts of the world [14,15,16], and the virome mapping in *Culex* mosquitoes is no exception. To date, there are at least four studies examining the virome of *C. tritaeniorhynchus* mosquitoes [7,15,17,18]. All four studies have provinces in China as collection sites, whereas, in Japan, where JEV circulation has been going on for roughly 1.5 centuries, no virome study of the mosquito vectors has ever been done.

With a bulk RNA sequencing approach, such as NGS, the combination of virome profiling and disease surveillance became feasible; together, they may improve the understanding of the virus community in mosquitoes. This study comprehensively describes the virome profiles of two JE major vectors, *C. tritaeniorhynchus* and *C. pseudovishnui*, in comparison to that of another *Culex* species, *Culex inatomii*, collected in several prefectures in Japan. Using NGS, 27 viral genomes were uncovered, 15 of which were newly identified. These results revealed mosquito viral diversity, including the disease surveillance target, the JE.

## 2. Materials and Methods

### 2.1. Mosquito Collection and Species Identification

**Figure 1:**
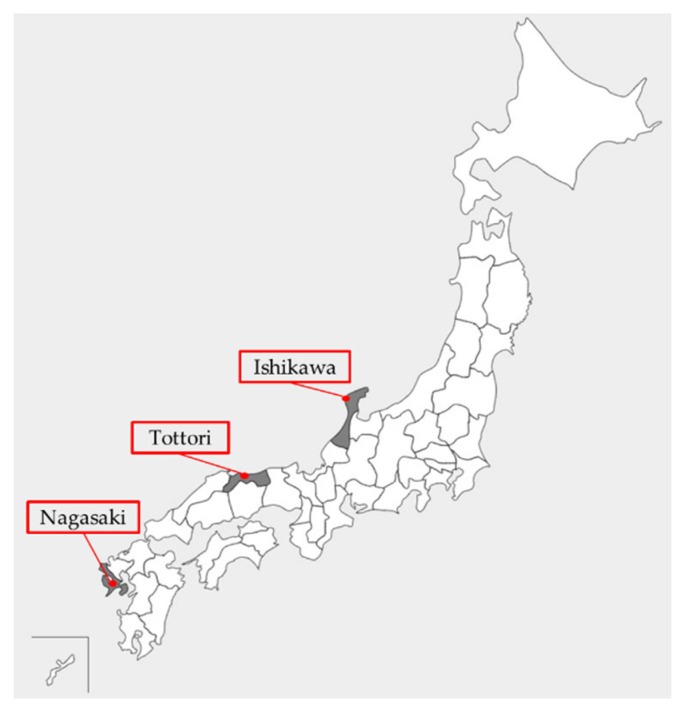
Map showing the mosquito collection sites in three prefectures of Japan: Ishikawa, Tottori, and Nagasaki.

**Figure 2:**
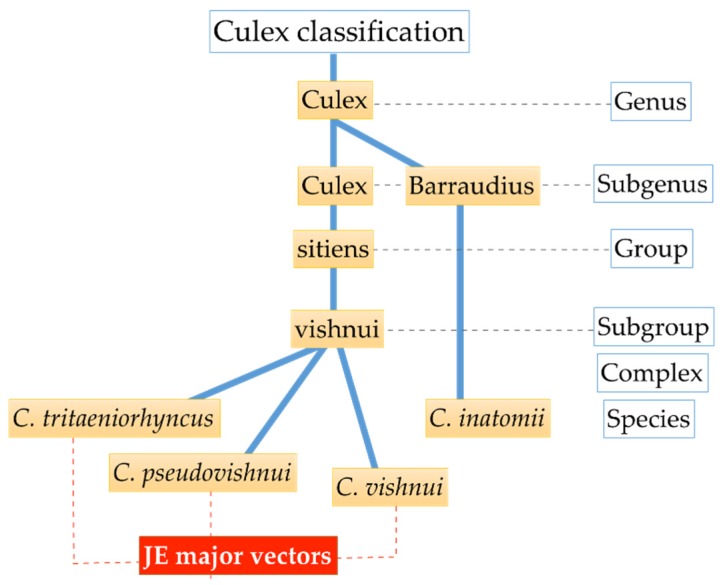
Classification of mosquitoes used in the present study. Three JE major vectors (*C*. *tritaeniorhynchus*, *C*. *pseudovishnui*, and *C*. *vishnui*) belong to the *C*. *vishnui* subgroup. Note that the *C*. *vishnui* subgroup and the outlier (*C*. *inatomii*) differ in the subgenus level.

Mosquito collections were conducted in three prefectures of Japan: Ishikawa, Tottori, and Nagasaki, using CDC miniature light traps (The John W. Hock Co., Gainesville, FL, USA) with CO_2_, sweep nets, and/or aspirators. The locations selected for mosquito collection were based on area descriptions with a presumed link to JEV circulation, such as near pigpens (Isahaya City in Nagasaki Prefecture (32°49′ N, 130°03′ E)), rice field areas (several cities in Ishikawa Prefecture (37°29′ N, 136°74′ E)), and a bird migration hotspot (Yonago waterbirds sanctuary in Tottori Prefecture (35°26′ N, 133°17′ E)), as shown in Figure 1. The identification of *Culex* spp. was according to established identification keys [19]. After identification, mosquitoes were sorted by species and stored at −80 °C until further experiment. Only *C. tritaeniorhynchus* and *C. pseudovishnui* mosquitoes, which belong to the *C. vishnui* subgroup, and *C. inatomii* as the outgroup were processed in this study. The biological classification (taxonomic rank) of *Culex* mosquitoes used in this study is shown in Figure 2.

### 2.2. Metagenomic Analysis of RNA Virome in Culex Mosquitoes

A total of 652 mosquitoes, sorted into 13 pools of 21 to 25 mosquitoes, were processed for viral metagenomic analysis (Table 1) based on a previously described method [20]. Briefly, mosquito pools were homogenized, filtered, and then nuclease treated before RNA extraction using ISOGEN II (Nippon Gene, Toyama, Japan). Subsequently, the sequencing libraries were constructed and the amount of cDNAs was quantified by quantitative real-time polymerase chain reaction (qRT-PCR). All libraries were sequenced on an NGS (Illumina MiniSeq, San Diego, CA, USA). CLC Genomics Workbench version 11 (Qiagen, Aarhus C, Denmark) was used for processing nucleotide reading datasets. Sequences with >500 bp in length were chosen for the *de novo* assembly approach and the resulting datasets were compared with other nucleotide and amino acid sequences by BLASTn and BLASTx on the NCBI database. Standard database (nucleotide collection (nr/nt)) and highly similar sequences (megablast) optimization were selected for the BLASTn program, while standard (1) genetic code and non-redundant protein sequences (nr) as he target database were used for BLASTx program. Sequences with an 0.0 e-value and high query cover with other viral species were considered as known, while ‘unknown’ or potential viral sequences were checked for open reading frames (ORFs) in GENETYX ver. 13 software (Genetyx Corp., Tokyo, Japan) and/or NCBI ORFfinder, with parameters of minimal ORF 75 nt in length, standard genetic code, and ‘any sense codon’ for ORF start. HHpred interactive server (https://toolkit.tuebingen.mpg.de/tools/hhpred) for protein homology detection and NCBI Conserved Domain Database version 3.17 with an expected value threshold of 1 × 10^−2^ were also used for viral sequence detection, particularly for unknown short contigs with ORF(s). Subsequently, the short viral contigs with unassembled overlaps were merged, and translated proteins were aligned with closely related viruses as a reference. Any gaps found were filled by RT-PCR and Sanger sequencing using the ABI PRISM 3130 Genetic Analyzer (Thermofisher, Waltham, MA, USA). To verify the assembly results, reads were mapped back to NGS datasets on CLC Genomic Workbench and reference sequences.

### 2.3. Virus Isolation

A total of 2323 mosquitoes were employed for virus isolation (Table 1). The mosquito C6/36 cell line derived from *Aedes albopictus* Skuse (European Collection of Authenticated Cell Cultures, Darmstadt, Germany) was used for virus isolation as described previously [21]. Pools of mosquitoes (consisted of 21–25 individual females per pool) were homogenized, filter sterilized, and inoculated onto cells followed by incubation for 2 h to allow virus adsorption. After the addition of fresh medium, cell cultures were incubated at 28 °C with the same conditions for about 7 days. The final harvest of supernatants was conducted after at least two blind passages and stored at −80 °C until use. To identify the isolated virus, supernatants from the final harvest were once again subjected to NGS using the MiniSeq system as described above and subsequently confirmed by RT-PCR. Total reads were obtained and then assembled de novo in CLC Genomics Workbench (Supplementary Table S1) Time course assay in C6/36 mosquito cells was also performed to ensure virus isolation.

### 2.4. Pools Confirmation Using RT-PCR and Sequence Analysis of Viral RNA

The confirmation of virus-positive pools was done by RT-PCR. Viral RNA was extracted from cell culture supernatants using ISOGEN II. RT-PCR was conducted using TaKaRa PrimeScript One-Step RT-PCR Kit version 2 (Takara Bio, Kusatsu, Shiga, Japan) according to the manufacturer’s instructions using gene-specific primers designed from detected contigs (Supplementary Table S2). PCR amplifications were carried out in a total volume of 10 μL consisting of 0.5 μL of template (cDNA), 0.5 μM of forward and reverse primers, 0.5 μL of PrimeScript One Step Enzyme Mix, 5 μL 2× One Step Buffer, and Milli-Q ddH2O to supplement the system. Thermal cycling was initiated with incubation at 50 °C for 30 s and denaturation step at 94 °C for 5 min; followed by 35 cycles of 94 °C for 30 s, 53 °C for 30 s, and 72 °C for 30 s. The amplified products were visualized by agarose gel electrophoresis followed by capillary sequencing using ABI PRISM 3130 Genetic Analyzer.

### 2.5. Viral Genome Characterization and Phylogenetic Analyses

A number of contigs and assembled putative viral sequences from NGS were finally obtained (see Section 2.2.). ORFs and amino acid translations were determined by GENETYX ver. 13 software (Genetyx Corp.) and NCBI Orffinder (https://www.ncbi.nlm.nih.gov/orffinder/) with a 300-nt minimum length. The genome structure was annotated using NCBI Conserved Domain Database version 3.17 (expected value threshold of 1 × 10^−2^) and the NCBI viral genome database as references.

To further characterize each viral isolate, the sequences were aligned by MAFFT version 7 (https://mafft.cbrc.jp/alignment/server/) employing G-INS-1 (slow) for a more accurate results and then trimmed by Gblocks version 0.91b (http://molevol.cmima.csic.es/castresana/Gblocks_server.html) [22] with a less stringent selection option (smaller final blocks, gap positions within the final blocks, and less strict flanking positions were allowed). Subsequently phylogenetic trees were constructed with MEGA version 6.06 using the maximum likelihood algorithm with 1000 bootstrap resampling. The substitution model and rates setting for the maximum likelihood tree were determined in advance by the best DNA/protein model searching tool. Viral genomes with less than or equal to 83% nucleotide identity to previously described viruses were identified as novel. The nucleotide sequence accession numbers are provided in Table 2 and Supplementary Table S3.

## 3. Results

### 3.1. RNA Virome Analysis by Metagenomic Sequencing

**Figure 3:**
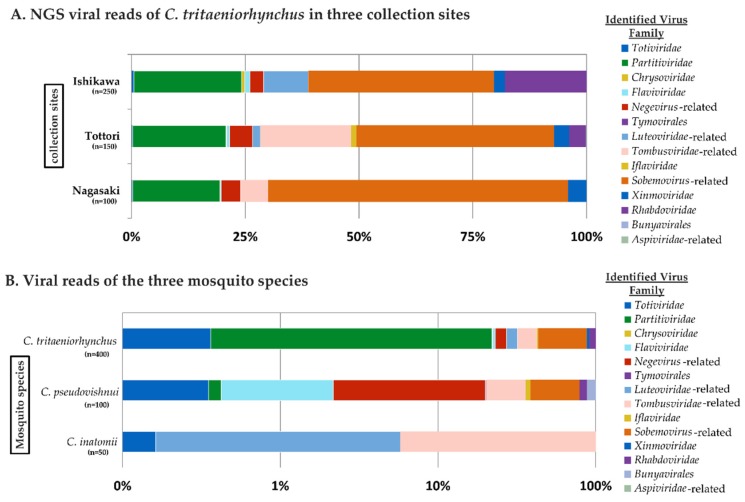
(**A**) Viral read ratio of *C. tritaeniorhynchus* identified virus families based on collection sites (Ishikawa, Tottori, and Nagasaki prefectures) along with the natural hosts of each virus family for better illustration. In general, *C. tritaeniorhynchus* collected from different locations showed the same virus families with variations in NGS reads. (**B**) The viral read ratio of all identified virus families based on mosquito species. The three mosquito species (*C. tritaeniorhynchus*, *C. pseudovishnui*, and *C. inatomii*) displayed different sets of virus families. Accordingly, the virome diversity of *C. tritaeniorhynchus* and *C. pseudovishnui* is richer than *C. inatomii*, which belongs to a different subgenus.

Viral metagenomic sequencing was performed on 13 pools of *Culex* mosquitoes, including both the well-known JE vector group (*C. vishnui* subgroup) and a unknown JE vector group (*C. inatomii*). The viromes of *C. tritaeniorhynchus* mosquitoes collected from three locations were almost identical, with seven out of nine viral families present across locations (Figure 3A). Regardless of the total number of samples used, it is interesting how virus families, such as Totiviridae, Partitiviridae, Flaviviridae, Iflaviridae, and Xinmoviridae, appeared concurrently, suggesting a robust relationship of the virome with the host, *C. tritaeniorhynchus*.

In comparison to the *C. tritaeniorhynchus* virome, the result of the *C. pseudovishnui* and *C. inatomii* viromes seemed to clarify the impact of the host taxon in the virome structure, as the three mosquito species have a different set of virus families (Figure 3B). Despite the differences, *C. tritaeniorhynchus* shared some virus families with the closely related species *C. pseudovishnui*, three families in total, including an unclassified virus family.

**Figure 4:**
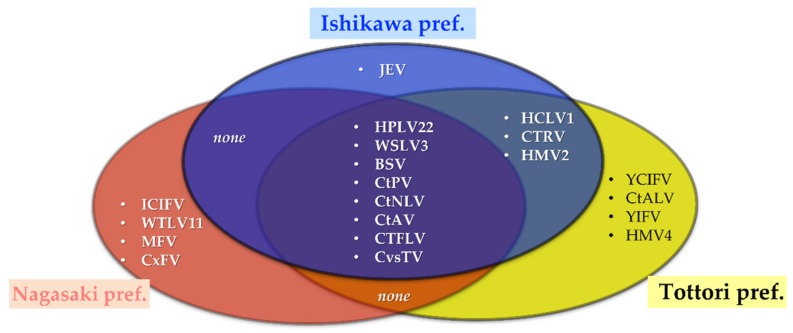
Venn diagram showing viruses identified from *C. tritaeniorhynchus* collected from three prefectures. Note the eight viruses in the overlapping region.

From a total of 27 identified viruses, 18, 11, and 3 viruses were identified from *C. tritaeniorhynchus*, *C. pseudovishnui*, and *C. inatomii*, respectively, suggesting viral abundance variation in each mosquito species (Figure 4, Table 3). Eight viruses were detected from all pools of *C. tritaeniorhynchus* mosquitoes collected from three prefectures, belonging to Partitiviridae, Flaviviridae, Totiviridae, and unclassified virus families, respectively (Figure 4, in the center of overlapping areas). The result showed corresponding patterns with the previous result of the virus family ratio, predisposing mosquito taxon over geographical location as the virome-shaping cause.

Culex pseudovishnui collected from one collection site in Nagasaki also displayed an interesting pattern in terms of shared viruses, as it shared three viruses with C. tritaeniorhynchus (Table 3). Being the outgroup, only three viruses were detected in C. inatomii, those belonging to Totiviridae and unclassified families.

Taken together, the viruses identified in this study comprised double-stranded RNA (dsRNA) viruses (Totiviridae, Partitiviridae, and Chrysoviridae families), positive-sense single-stranded RNA [(+)ssRNA] viruses (Flaviviridae and Iflaviridae families, and unclassified groups), and negative-sense ssRNA [(−)ssRNA] viruses (Xinmoviridae and Rhabdoviridae families, and unclassified groups). Further details of each virus are described below.

### 3.2. Dataset Breakdown of Mosquito RNA Viruses

The metagenome sequencing method uncovered a wide range of viruses from a total of 652 mosquitoes grouped into 13 pools (Table 1). In total, BLAST analyses of NGS viral reads identified 27 RNA viral genomes, 15 of which were newly described (Table 2, Supplementary Table S3). All characterized viruses in this study were phylogenetically within an order and related or belonged to viral families listed in the International Committee on Taxonomy of Viruses.

#### 3.2.1. dsRNA Viruses

##### Totiviridae

**Figure 5:**
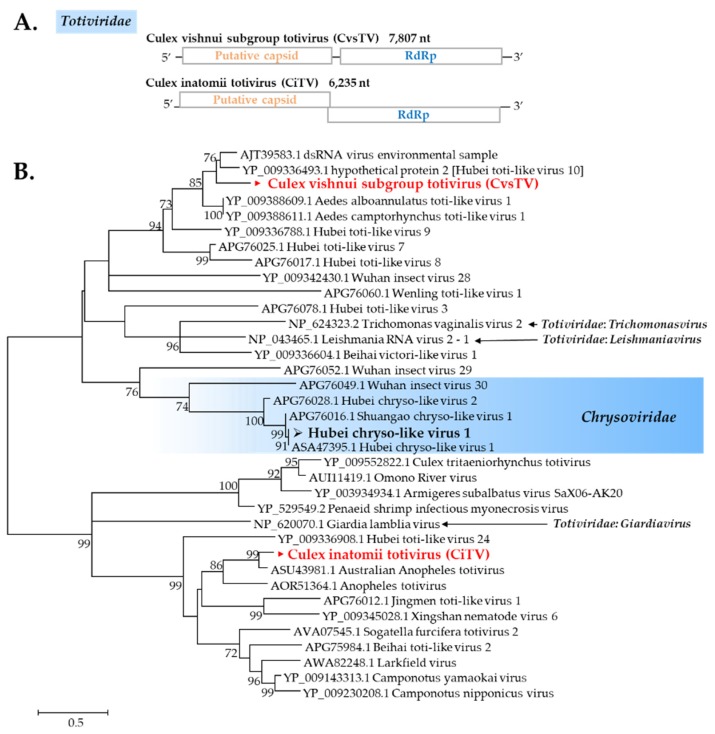
(**A**) Genome organization of two novel totiviruses found in this study. Note the difference in the overlapping region. (**B**) Maximum likelihood phylogeny of Totiviridae and Chrysoviridae clade using ~123 amino acid sequences of RdRp conserved domains selected by Gblocks. Values on branches indicate bootstrap support based on 1000 bootstrap replicates. Bootstrap values <70 are not shown. ► indicates novel viruses and ➢ indicates a new strain identified in this study.

Totiviruses are known as a group of dsRNA viruses that infect fungi, protozoa, or invertebrates [23]. In this study, most mosquito pools have a totivirus sequence present. *Culex vishnui* subgroup mosquitoes have the exact same totivirus beyond collection sites (designated as *Culex vishnui* subgroup totivirus, CvsTV), whereas *C*. *inatomii* mosquitoes have a different totivirus (designated as *Culex inatomii* totivirus, CiTV) (Figure 5A). CvsTV was highly distributed in the pools tested, and it was present in *Culex vishnui* subgroup pools (Table 3). CvsTV presented a close relationship with dsRNA virus environmental sample (Figure 5B), whereas CiTV formed a cluster with Australian *Anopheles* totivirus.

##### Chrysoviridae

Chrysoviruses are known to infect fungi [24], but recent studies have revealed an association with insects. Hubei chryso-like virus 1 (HbCl-1) was described previously in mosquitoes [25]. In this study, a new strain of this viral sequence was found in most pools of *C*. *tritaeniorhynchus* originating from Ishikawa (Figure 5).

##### Partitiviridae

**Figure 6:**
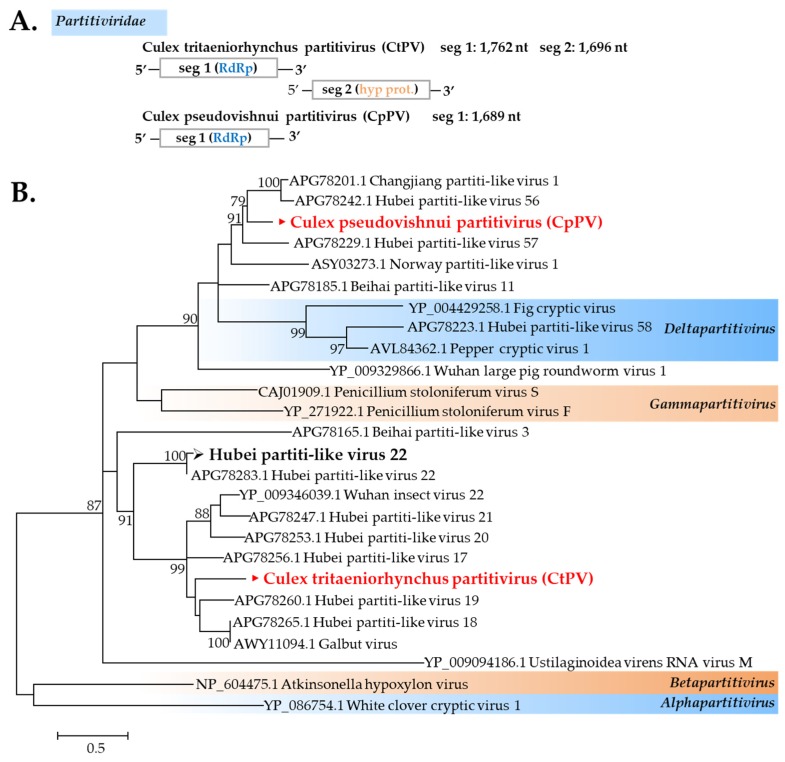
(**A**) Genome organization of two novel partitiviruses found in this study. (**B**) Maximum likelihood phylogeny of the Partitiviridae using ~163 amino acid sequences of RdRp conserved domains selected by Gblocks. Values on branches indicate bootstrap support based on 1000 bootstrap replicates. Bootstrap values <70 are not shown. Viruses identified in this study are in bold, with ► indicating novel viruses and ➢ indicating a new strain identified in this study.

Partitiviruses are widely known to be associated with plants and fungi; however, recent studies have suggested that their known host range might be expanded, and some members in this genus were discovered in arthropods [26,27]. In this study, two partitivirus sequences were found in *C*. *tritaeniorhynchus*: New strains of Hubei partiti-like virus 22 and a novel partitivirus designated as *Culex tritaeniorhynchus* partitivirus (CtPV). *Culex pseudovishnui* has a different novel partitivirus named *Culex pseudovishnui* partitivirus (CpPV). CtPV formed a cluster with Hubei partiti-like virus 19, Hubei partiti-like virus 18, and Galbut virus, whereas CpPV displayed a correlation with Hubei partiti-like virus 56 and Changjiang partiti-like virus 1 (Figure 6).

#### 3.2.2. (+)ssRNA viruses

The majority of viruses identified in this study were (+)ssRNA viruses. As many as 15 (+)ssRNA viral genomes were identified. Some were previously described as ISVs, some were novels, and one was a mosquito-borne arbovirus.

##### Flaviviridae

**Figure 7:**
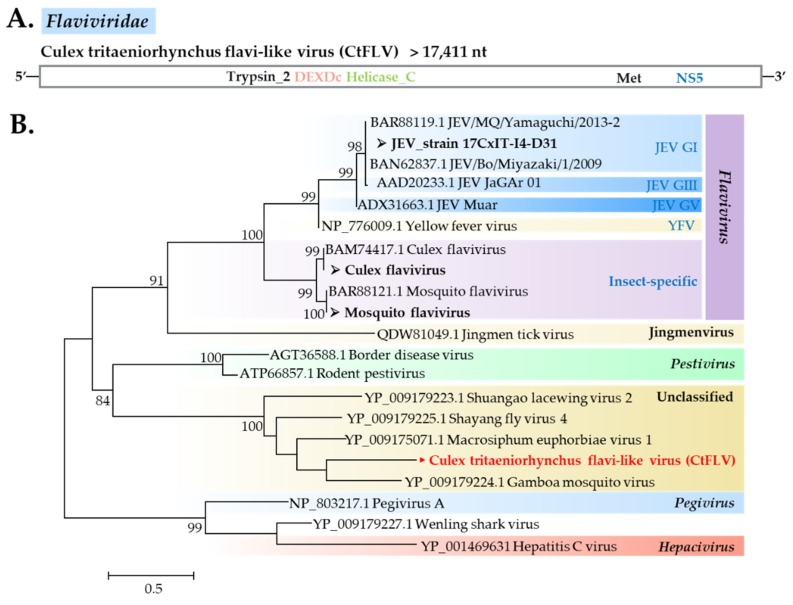
(**A**) Genome organization of a novel flavi-like virus found in this study. (**B**) Maximum likelihood phylogeny of Flaviviridae using ~297 amino acid sequences of the NS5 region. Values on branches indicate bootstrap support based on 1000 bootstrap replicates. Bootstrap values <70 are not shown. Viruses identified in this study are in bold, with ► indicating novel viruses and ➢ indicating a new strain identified in this study.

This virus family has humans and mammals asits natural host, which makes virus discovery of this family noteworthy. Flaviviridae members found by NGS are mosquito flavivirus, *Culex tritaeniorhynchus* flavi-like virus (CtFLV), and JEV. A strain of mosquito flavivirus, described as Yamadai flavivirus, was found in two *C*. *pseudovishnui* pools. CtFLV belonged to an unclassified flavivirus clade together with viruses discovered from arthropods: Gamboa mosquito virus, Macrosiphum euphorbiae virus 1, Shayang fly virus 4, and Shuangao lacewing virus 2 (Figure 7), based on the NS5 region.

**Figure 8:**
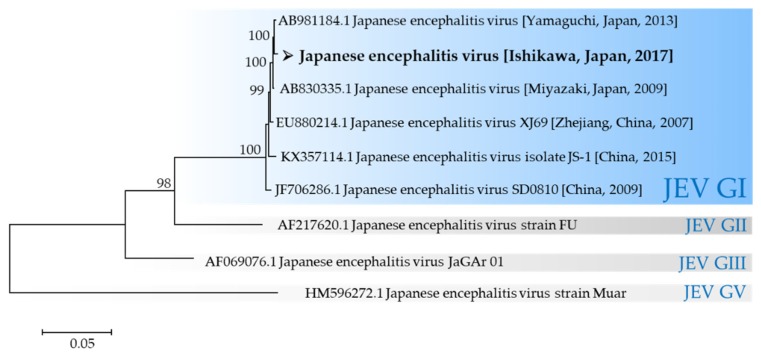
Maximum likelihood phylogeny of JEV using ~10,995 nucleotide sequences. Values on branches indicate bootstrap support based on 1000 bootstrap replicates. Bootstrap values <70 are not shown. ➢ indicates a new strain identified in this study.

JEV strain 17CxIT-I4-D31 was successfully characterized as genotype I based on envelope region, and it was isolated from a *C*. *tritaeniorhynchus* sample from Ishikawa. Based on the evolutionary tree (Figure 8), JEV identified in this study was closely related to isolates from relatively distant areas, such as JEVs detected in Miyazaki Prefecture in 2009, Yamaguchi Prefecture in 2013, and Zhejiang, China in 2007. As widely known, there are five genotypes of JEV in the world; currently, genotype I is the predominant genotype [28,29].

##### Negevirus-Related Viruses

**Figure 9:**
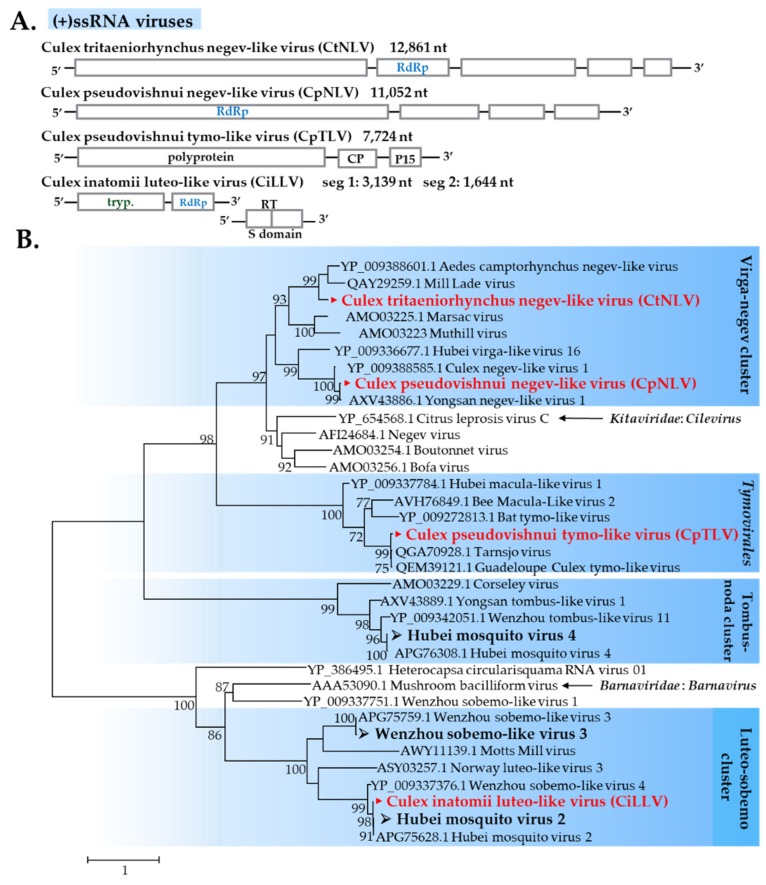
(**A**) Genome organization of four novel (+)ssRNA viruses found in this study. (**B**) Phylogenetic analysis of several (+)ssRNA virus families was performed with the maximum likelihood method using ~4396 amino acid sequences of the RdRp region. Node bootstraps were calculated with 1000 replicates. Bootstrap values <70 are not shown. Viruses identified in this study are in bold, with ► indicating novel viruses and ➢ indicating a new strain identified in this study.

Negevirus was recently described as a diverse group of ISVs isolated from mosquitoes and phlebotomine sandflies [30]. In this study, two novel negevirus-like sequences were found in *C*. *vishnui* subgroup mosquitoes. A novel virus was identified from all pools of *C*. *tritaeniorhynchus* and designated as *Culex tritaeniorhynchus* negev-like virus (CtNLV). Another novel virus was identified from *C*. *pseudovishnui* and designated as *Culex pseudovishnui* negev-like virus (CpNLV). Phylogenetic analysis revealed that CtNLV formed a cluster with Mill Lade virus and *Aedes camptorhynchus* negev-like, whereas CpNLV formed a cluster with Yongsan negev-like virus (Figure 9).

##### Tymovirales

Tymovirales is an order of non-segmented (+)ssRNA viruses, which currently includes five families. Most members of the order infect plants, but a few species are from plant pathogenic fungi [31]. Generally, members of the family Tymoviridae have plants as their natural hosts with insects as a mechanical vector. Studies have recently discovered tymo-related viruses, such as Tarnsjo virus and Guadeloupe Culex-tymo-like virus in mosquitoes, thus implying the relationship in nature. A tymo-like virus found in this study was detected in two pools of *C*. *pseudovishnui* and designated as *Culex pseudovishnui* tymo-like virus (CpTLV). CpTLV formed a cluster with two recently identified viruses: Tarnsjo virus and Guadeloupe Culex-tymo-like virus (Figure 9).

##### Luteoviridae-Related Viruses

Luteoviruses are a family of viruses commonly known to include many economically important plant pathogens [32]. Recently, luteo-like viruses (related but phylogenetically divergent compared to recognized members) have been discovered in diverse invertebrates, such as dragonflies, spiders, and mosquitoes, indicating that this group of viruses has wider range than previously known [27,33]. In this study, three known luteo-like viruses were identified in *C*. *tritaeniorhynchus* and/or *C*. *pseudovishnui*: Wenzhou tombus-like virus 11 (WTLV11), Hubei mosquito virus 2 (HMV2), and Hubei mosquito virus 4 (HMV4) (Figure 9). A novel luteo-like virus designated as *C. inatomii* luteo-like virus (CiLLV) was identified in *C*. *inatomii*. Based on the RdRp region, CiLLV was closely related to HMV2 (Figure 9); likewise, the nucleotide sequence of segment 1 and segment 2 displayed similar results (Supplementary Figure S1).

##### Sobemovirus-Related Viruses

For sobemoviruses, plants are their sole natural host. However, bulk RNA sequencing has increased the scope of their host range, with some viruses that belong to this group found in vertebrates and arthropods [33]. In this study, two known sobemo-like viruses, Wenzhou sobemo-like virus 3 (WSLV3) and bat sobemovirus, were found in all pools of *C*. *vishnui* subgroup mosquitoes but not in the *C*. *inatomii* library.

**Figure 10:**
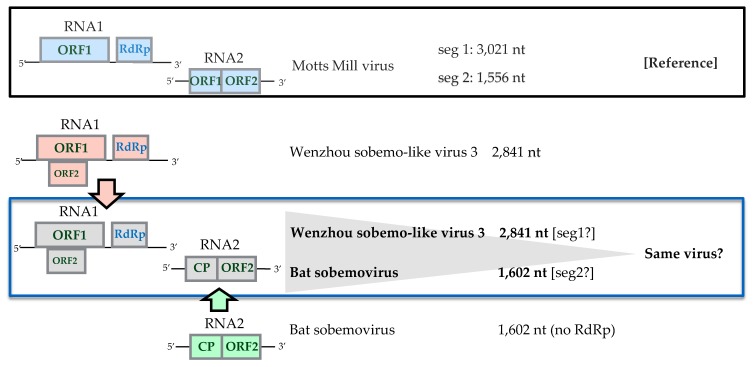
Genome organization of two viruses found in this study. The appearance of two viruses in the same mosquito pools has inferred the possibility of the two sequences being the same virus. Motts Mill virus (accession number MH384280) is used as reference genome.

WSLV3 was reported to have 2841 bp nucleotides [33], shorter than the average sobemo-like viruses that have 4000 to 5000 bp nucleotides. In contrast, only the coat protein of bat sobemovirus was submitted by Dacheux et al. [34] without any other segments nor the polymerase region. Furthermore, multiple appearances of these two viruses in the same mosquito pools have inferred the possibility of the two being the same virus (as illustrated in Figure 10), although more studies are required to confirm this finding.

##### Iflaviridae

**Figure 11:**
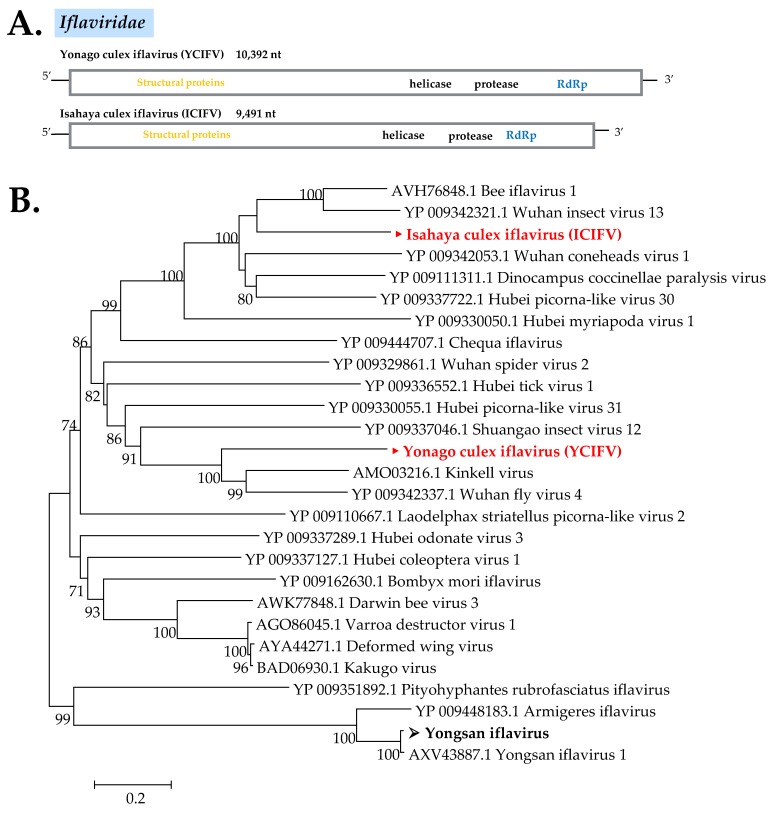
(**A**) Genome organization of two novel iflaviruses found in this study. (**B**) Phylogenetic analysis of Iflaviridae was performed with the maximum likelihood method using ~971 amino acid sequences of RdRp conserved domains selected by Gblocks. Node bootstraps were calculated with 1000 replicates. Bootstrap values <70 are not shown. Viruses identified in this study are in bold, with ► indicating novel viruses and ➢ indicating a new strain identified in this study.

Iflaviruses are widely known as a group of picorna-like viruses that infect arthropods [35]. In this study, three iflaviruses were found: A novel iflavirus designated as Isahaya Culex iflavirus (ICIFV), which was detected in most pools of *C*. *tritaeniorhynchus* and *C*. *pseudovishnui* collected in Nagasaki; another strain of Yongsan iflavirus; and a novel iflavirus designated Yonago Culex iflavirus (YCIFV), which was detected from *C*. *tritaeniorhynchus* pools from Tottori. The phylogenetic tree displayed ICIFV as being in the same cluster with Bee iflavirus 1 and Wuhan insect virus 13, whereas YCIFV formed a cluster together with Kinkell virus and Wuhan fly virus 4 China (Figure 11).

#### 3.2.3. (−)ssRNA Viruses

Five putative (−)ssRNA viruses, including major taxonomic categories, were discovered. Among these, one virus is a known mosquito virus, whereas the remaining four viruses are newly described in this study.

##### Xinmoviridae

**Figure 12:**
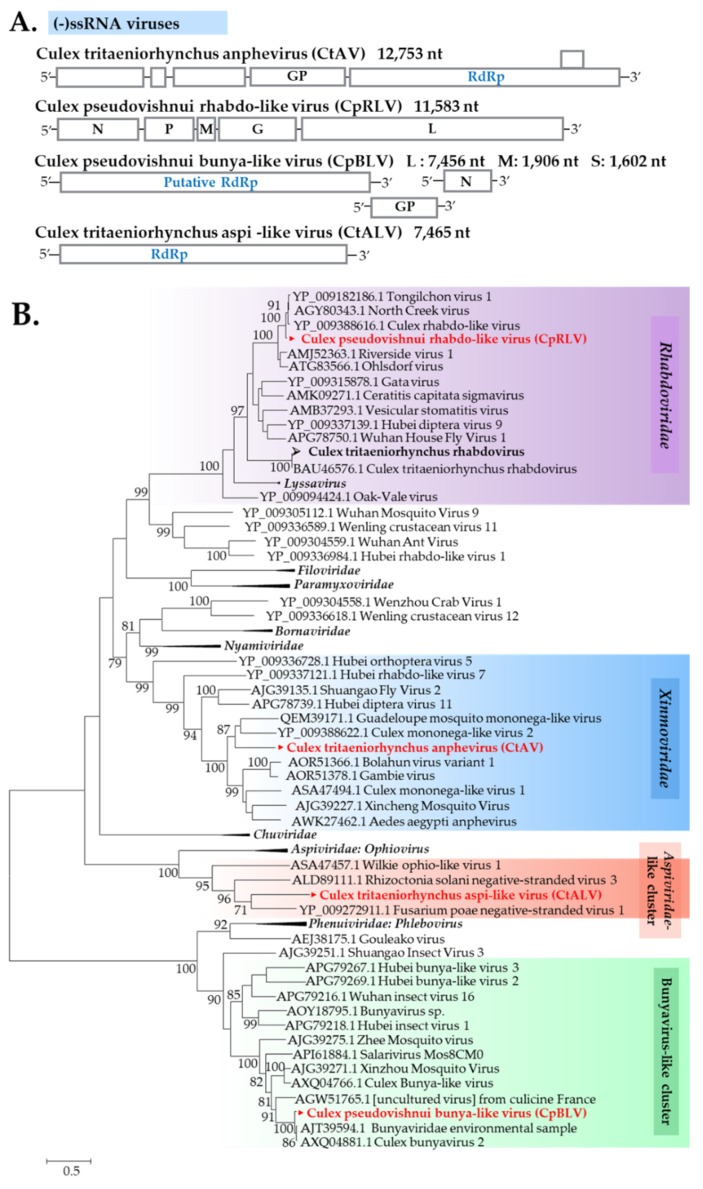
(**A**) Genome organization of four novel (−)ssRNA viruses found in this study. (**B**) Phylogenetic analysis of several (−)ssRNA virus families was performed with the maximum likelihood method using ~4556 amino acid sequences of the RdRp region. Node bootstraps were calculated with 1000 replicates. Bootstrap values <70 are not shown. Viruses identified in this study are in bold, with ► indicating novel viruses and ➢ indicating a new strain identified in this study.

The recently updated classification of Mononegavirales taxon has a new family named Xinmoviridae, established for the floating genus *Anphevirus* [36,37]. In this study, a new member of the virus family, designated as *C. tritaeniorhynchus* anphevirus (CtAV), was found in most pools of *C*. *tritaeniorhynchus*. Phylogenetic analysis showed a close relationship of CtAV with Guadeloupe mosquito mononega-like virus and *Culex mononega*-like virus 2 (Figure 12).

##### Rhabdoviridae

Rhabdoviruses are a diverse set of (−)ssRNA viruses known to infect both animals and plants [38], with frequent host switching during their evolution history [39]. Two rhabdoviruses were detected in this study: *Culex tritaeniorhynchus* rhabdovirus was found in *C*. *tritaeniorhynchus* only from Ishikawa and Tottori, whereas a novel virus designated as *Culex pseudovishnui* rhabdo-like virus (CpRLV) was solely identified in *C*. *pseudovishnui*. CpRLV displayed a close relationship with Tongilchon virus (Figure 12).

##### Bunyavirales

Bunyavirales is an order of segmented (−)ssRNA viruses, which currently includes more than 10 families [40,41]. In this study, sequences related to bunyavirus L and M genes were detected from two *C*. *pseudovishnui* pools. The new bunya-like virus was tentatively named *C. pseudovishnui* bunya-like virus (CpBLV). The phylogenetic tree based on the RdRp sequences encoded by the L segment revealed CpBLV as being in the same clade as RdRp of the Bunyaviridae environmental sample and Culex bunyavirus 2, which possibly belong to the Phenuiviridae family (Figure 12). Phylogenetic analysis of M and S segments of CpBLV were also provided (Supplementary Figure S2)

##### Aspiviridae-Related Viruses

In this study, a virus related to the Aspiviridae (formerly called Ophioviridae) family, was identified, which was designated *Culex tritaeniorhynchus* aspi-like virus (CtALV). CtALV was detected in a *C*. *tritaeniorhynchus* pool collected in Tottori. The phylogenetic tree showed that CtALV is closely related to Wilkie ophio-like virus 1 detected from *A*. *camptorhynchus* mosquitoes in Australia [33] and ophiovirus-related viruses of plant pathogenic fungi [42] (Figure 12).

### 3.3. Attempt to Isolate Viruses Using a Mosquito Cell Line

C6/36 cells derived from *A*. *albopictus* were used in this study to isolate the identified viruses (Table 1) and to disclose any latent viruses undetected by NGS. After three blind passages to propagate viruses, supernatants were harvested before being subsequently examined with time course assay for validation of the virus isolation. Through this method, one medically important virus and five insect-related viruses were successfully isolated by C6/36 cells: JEV, mosquito flavivirus, *Culex* flavivirus (CxFV), and a novel iflavirus called YCIFV.

Although the cell line used was mosquitoes, the isolated viruses were not limited to ISVs. Moreover, the fact that not every ISV was isolatable illustrated the cell line had variable sensitivity to virus infection. Lineages of the C6/36 cell line have been widely used to study the relationship between arboviruses and mosquito vectors [43] and they have been shown to be susceptible to a wide range of arboviruses, partially due to the lack of a functional RNA interference response [44].

Three blind passages of virus isolation in mosquito cell lines amplified two viruses not detected by NGS: CxFV and mosquito flavivirus in the Nagasaki group’s *C*. *tritaeniorhynchus*. The fact that those undetected viruses belong to the *Flavivirus* genus, which contains many medically important viruses, highlighted the sense that, in arbovirus surveillance, the method of virus amplification through mosquito cell lines should not be overlooked.

### 3.4. Pools Confirmation and Additional Individual Screening

Using the NGS result as the lead, primers were designed and RNA viruses in pools were confirmed by RT-PCR; thus, the virome of each mosquito species and collection sites can be analyzed and putative seasonal changes of virome composition can also be portrayed (Supplementary Figure S3). There were two patterns observed in seasonal virome composition: Continuous presence (detected in every month during mosquito emergence of early summer to late autumn) and occasional presence (irregular detection). To establish the proportion of highly prevalent viruses in mosquitoes, additional data of individual infection rates were provided. Results showed percentages of infectivity that ranged from 4% to 58% (Supplementary Table S4), suggesting the active circulation of viruses infecting most of the mosquitoes in the population. Nevertheless, further studies over a longer period of time (3–5 years) would be required to confirm the seasonal changes.

## 4. Discussion

It is estimated that approximately 67,900 JE cases typically occur annually in 24 JE-endemic countries [1], including Japan. A survey on the natural infection of JEV in humans and horses indicated that JE remains prevalent in Japan [45,46] and *C*. *tritaeniorhynchus* belonging to the *vishnui* subgroup is the main vector in Japan [47]. This study successfully combined mosquito-borne arbovirus surveillance with virome profiling in major JE vectors collected in three prefectures in Japan.

The virome of three *Culex* mosquito species, *C*. *tritaeniorhynchus* and *C*. *pseudovishnui* renowned as important vectors for JEV in Asia and *C*. *inatomii* as the non-vector species, was analyzed to better understand arbovirus–ISV interaction. The abundance and diversity of possible ISVs constituted a significant component of *Culex* mosquito viromes; for example, the virome of *Culex* spp. in Zambezi, Mozambique was dominated (88.5%) by them [48], whereas a study of the *C. tritaeniorhynchus* virome in Hubei, China also showed a conformable predominance (88%) of insect viruses [18]. In line with the reckonings, the result displayed a practically similar constitution, as much as 84%, 82%, and 100% of viruses were insect viruses, or had a high correlation with the mosquito virome previously identified, in *C*. *tritaeniorhynchus*, *C*. *pseudovishnui*, and *C*. *inatomii*, respectively (Supplementary Figure S4), implying a consistent virome composition in *Culex* mosquitoes, with slight variations possibly associated with the number of samples used.

Metagenomic sequencing results suggested a distinct virus distribution depending on the mosquito taxonomy. Here, it was shown that two mosquitoes belonging to the same subgroup, *C*. *tritaeniorhynchus* and *C*. *pseudovishnui*, shared a number of virus families (Totiviridae, Partitiviridae, Flaviviridae, Iflaviridae, and Rhabdoviridae), whereas another *Culex* belonging to a different subgenus, *C*. *inatomii*, shared only one virus with mosquitoes collected in the same area. This finding corresponds to a study comparing viral diversity in some species of mosquitoes, which showed correlation between mosquito species and their virome structures [49].

Recurring detection of certain predominant viruses from one mosquito species, *C*. *tritaeniorhynchus*, originated from various collection sites, and time has corroborated the taxon impact on virome shaping even more. The absence of these predominant viruses in other species could imply disparity in host susceptibility, although this would need further clarification.

Not only does this study display a pattern of host taxon as one of the virome-shaping factors, but it also reveals another point—that some viruses could be detected in discrete *Culex* mosquito species inhabiting the same area. This phenomenon was vividly demonstrated by detection of HMV4, which was detected from the two mosquito species (*C*. *tritaeniorhynchus* and *C*. *inatomii*) collected from the same sites in Tottori. In some way, particular viruses present in this study were also discovered in geographically distant localities, such as HMV2 and WSLV3, which were already detected in *C*. *tritaeniorhynchus* in China [17]. In the beginning, virus predominance in *Culex* sp. mosquitoes has long been speculated, before nucleotide identities (99% in both viruses) remarked an untypical limited genetic transformation despite the geographical distance. Again, this correlated with a previous finding that some viruses infected hosts over a wide geographical area and were possibly introduced by windblown, cyclones, or humans rather than the spread of mosquitoes across continents over time [33,50,51,52]. Clearly, however, more extensive studies need to be undertaken to determine if these virome patterns are consistent beyond mosquito species and geographic regions.

Aside from the patterns observed, this study also discusses the viral association with hosts and possible interrelation with other coexisting viruses. The arbovirus transmitted by mosquitoes detected in the study was JEV, which was identified in *C*. *tritaeniorhynchus* from Ishikawa Prefecture. No other virus was considered as arbovirus based on their closest related viruses or phylogenetic tree. An interesting aspect exhibited from virus isolation might be that another flavivirus, CxFV, was not detected in the mosquito homogenate before virus isolation. Nagasaki Prefecture, where CxFV isolates were collected, is a reputable JE-endemic area in Japan, as JEV detection is frequent in annual disease surveillance of amplifiers and vectors. JEV was not isolated nor identified in 2017 Nagasaki samples, thus making it difficult to decipher the interrelation between the flaviviruses. Although an in vitro study by Kuwata et al. [53] implied that JEV superinfection might be harmful to CxFV-infected *C*. *tritaeniorhynchus* mosquitoes, this study was unable to demonstrate the correlation between CxFV occurrence and the detection or otherwise of JEV.

The only collection site where JEV was identified is Monzenmachi-toge, Ishikawa Prefecture, where the landscape is a vast rice field surrounded by forest with the river and sea in close proximity. The result was supported by annual disease surveillance conducted in September 2017 by the Japan National Institute of Infectious Disease (2017) [54], which has indicated that 50% to 60% of swine sera had JE antibodies, although, in the previous 2 years (2015 and 2016), the range was only between 0% and 10%. An increase of JEV antibodies in swine sera contributed greatly to JEV detection in mosquito, as swine plays a major role as a JEV amplifier [55].

Phylogenetic analysis of the complete genome showed that the isolated JEV strain 17CxIT-I4-D31 was closely related to JEV isolates detected in Yamaguchi in 2013 rather than those from Ishikawa previously or other surrounding areas, suggesting that JEV may be transported from west Japan into central Japan. The phenomenon of this virus spread has also occurred in Getah virus (genus *Alphavirus*, family Togaviridae). A study reported the virus being transported in the same direction from west Japan into central Japan, with possible routes of transportation, such as bird migration and windblown mosquitoes [56].

Detection of JEV in Ishikawa, where other ISVs are circulating simultaneously (Supplementary Figure S3), could signify the natural occurrence of coexistence of the virome. However, this must be interpreted carefully, as this study used mosquito pools instead of individual mosquitoes.

Although this work used mosquitos as the sample, surprisingly, viral hosts other than mosquitos appeared from the result. As mentioned previously, where identified viruses were either insect specific or human arbovirus, another virus host, which is a disease vector, emerged. Bat sobemovirus (named Bordeaux sobemovirus) was first identified from pooled organs (lungs, liver, and brain) of *Pipistrellus pipistrellus*, an insectivorous bat, in France in 2009 [34]. Yet, it found its way into the *C*. *vishnui* subgroup mosquitoes in Japan. This finding is not novel, as other studies similarly found the linkage between the bat and arthropod through the virome [57,58]. However, it should be noted that the bat might have a key role in the sylvatic cycle of flaviviruses [59]; additionally, there has been evidence of JEV presence in the bat [60,61]. Therefore, substantial appearances of bat sobemovirus in all libraries of *C*. *vishnui* subgroup mosquitoes may strengthen the possible relationship between bat and JE mosquito vectors.

Nevertheless, the bat sobemovirus nucleotide sequence identified by Dacheux et al. (2014) [34] was a viral coat protein with no polymerase or other segments. In this study, bat sobemovirus sequences were found in pools where WSLV3 was also detected. More studies are required to clarify the connection between the two viral sequences; however, the trace this study gave has indicated the possibility of the two being the same virus.

Importantly, this mosquito virome study was carried out using metagenomic sequencing in combination with mosquito cell line virus isolation. Efforts have been put into metagenomic analysis using NGS for disease surveillance, as the method allows the simultaneous identification of viruses from a single mosquito in a single reaction [62]. However, NGS methods have disadvantages compared to other molecular methods of virus detection, as they are less sensitive than qRT-PCR for the detection of samples with low virus titers, particularly when using mosquitoes as samples.

The combination of high-throughput sequencing and virus isolation in this study was meant to retrieve the complete mosquito virome, including underlying viruses that exist at very low copy numbers, whereupon the results can be interpreted. C6/36 cells were selected, as they are easy to maintain and highly permissive to numerous arboviruses [63], although the limitation is that they may fail to accurately model mosquito–arbovirus interactions [44], and the output given for this virome identification is of importance, as they successfully uncovered viruses undetected by preceding NGS. The detection of two insect-specific flaviviruses (ISF), CxFV and MFV, in only the third supernatants should not be overlooked. This is because they belong to a medically important virus genus and thus may imply potentially harmful viruses persisting at low copy numbers, undetected by NGS.

Finally, complete analysis was performed on the virome profile of major JE vectors in Japan, including an arbovirus, ISVs, and other viruses. This is the first report on the *Culex* mosquito virome study in Japan. In addition to JEV and several known ISVs, other novel viral sequences were identified from these mosquitoes. Notwithstanding, further studies will be required to confirm the real host of the identified viruses as the metatranscriptomic studies may also include non-mosquito RNA. By studying one of the medically important disease vectors, this study offers important insights into the ecology and evolution of mosquito viromes consisting of both arbovirus and insect viruses.

## Figures and Tables

**Table 1:** The profile of mosquito samples employed in metagenomic analysis and virus isolation.

Mosquito Species	Mosquito Collection Site	GPS Coordinates	Collection Month	Methods				
				Metagenomic Analysis		Virus Isolation		
				No. of Pools Tested	No. of Female Mosquitoes Tested	No. of Pools Tested	No. of Female Mosquitoes Tested	JEV-Positive Pools
*C. tritaeniorhynchus*	Monzen-machi, Wajima city, Ishikawa	37°29′ N, 136°74′ E	Jun–Oct	5	250	12	300	1
			2017					
*C. tritaeniorhynchus*	Yonago waterbirds sanctuary, Yonago city, Tottori	35°26′ N, 133°17′ E	Jun–Sep	3	150	8	200	0
			2017					
*C. tritaeniorhynchus*	Teramine farm, Isahaya city, Nagasaki	32°49′ N, 130°03′ E	Aug	2	100	54	1,350	0
			2017					
*C. pseudovishnui*	Teramine farm, Isahaya city, Nagasaki	32°49′ N, 130°03′ E	Aug	2	100	17	421	0
			2017					
*C. inatomii*	Yonago waterbirds sanctuary, Yonago city, Totttori	35°26′ N, 133°17′ E	Jun	1	52	2	52	0
			2017					
Total				13	652	93	2323	1

**Table 2:** Virome identification result (representative genomes or RdRp segments only).

Virus Category	VIRUS TAXON	Virus Name	Closely Related Viruses (Accession no.)	Blastn			Blastx			Location ^†^/Species Origin ^‡^	Segment	Accession no. [This Study]	Complete CDs ^¶^
				Identity (%) *	Query Cover (%)	e-Value	Identity (%) *	Query Cover (%)	e-Value				
dsRNA	Totiviridae	*Culex vishnui* subgroup totivirus (CvsTV)	dsRNA virus environmental sample (AJT39583)	-	-	-	46	39	0.0	Japan/CtrCps	-	LC514295	Y
	Totiviridae	*Culex inatomii* totivirus (CiTV)	Australian *Anopheles* Totivirus (ASU43981)	-	-	-	61	37	0.0	Tottori/Cnt	-	LC514398	Y
	Partitiviridae	*Culex pseudovishnui* partitivirus (CpPV)	*Hubei partiti*-like virus 56 (APG78242)	-	-	-	56	85	0.0	Nagasaki/Cps	1	LC514399	Y
	Partitiviridae	Hubei partiti-like virus 22 (HPLV22)	(APG78283)	99	98	0.0	99	98	0.0	Tottori/Ctr Nagasaki/Ctr	1	LC514400 LC514401	Y Y
	Partitiviridae	*Culex tritaeniorhynchus* partitivirus (CtPV)	*Hubei partiti*-like virus 19 (APG78260)	-	-	-	49	84	1 × 10^−170^	Japan/Ctr	1	LC514402	Y
	Chrysoviridae	Hubei chryso-like virus 1 (HCLV1)	(ASA47395)	84	99	0.0	-	-	-	Ishikawa/Ctr	1	LC514396	Y
(+)ssRNA	Flaviviridae	*Culex tritaeniorhynchus* flavi-like virus (CtFLV)	Shayang fly virus 4 (YP_009179225)	-	-	-	41	71	0.0	Japan/Ctr	-	LC514290	N
	Flaviviridae	Mosquito flavivirus (MFV)	(BAR88121)	98	99	0.0	99	93	0.0	Nagasaki/Ctr Nagasaki/Cps	-	LC513840 LC513841	Y Y
	Flaviviridae	Japanese Encephalitis Virus (JEV)	(AB981184)	99	100	0.0	99	97	0.0	Ishikawa/Ctr	-	LC513838	Y
	Flaviviridae	*Culex* Flavivirus (CxFV)	(BAM74417)	97	100	0.0	99	93	0.0	Nagasaki/Ctr	-	LC513839	Y
	Negevirus-related	*Culex* tritaeniorhynchus negev-like virus (CtNLV)	Mill Lade virus (QAY29259)	-	-	-	47	22	0.0	Japan/Ctr	-	LC507097	Y
	Negevirus-related	Culex pseudovishnui negev-like virus (CpNLV)	Yongsan negev-like virus 1 (AXV43886)	-	-	-	87	73	0.0	Nagasaki/Cps	-	LC512731	Y
(+)ssRNA	Tymovirales	*Culex pseudovishnui* tymo-like virus (CpTLV)	Tarnsjo virus (QGA70928)	79	76	0.0	74	84	0.0	Nagasaki/Cps	-	LC512732	Y
	Luteoviridae-related	Hubei mosquito virus 2 (HMV2)	APG75628	99	99	0.0	100	34	0.0	Ishikawa/Ctr Tottori/Ctr	1 1	LC513829 LC513830	Y Y
	Luteoviridae-related	*Culex inatomii* luteo-like virus (CiLLV)	Hubei mosquito virus 2 (APG75628)	83	99	0.0	-	-	-	Tottori/Cnt	1	LC513833	Y
	Tombusviridae-related	Hubei mosquito virus 4 (HMV4)	(APG76308)	91	82	0.0	-	-	-	Tottori/Ctr Nagasaki/Cnt	-	LC512733 LC512734	Y N
				90	98	0.0	-	-	-	Tottori/Ctr Nagasaki/Cnt	-	LC512735 LC512736	N N
	Tombusviridae-related	Wenzhou tombus-like virus 11 (WTLV11)	(YP_009342051)	99	99	0.0	100	28	0.0	Nagasaki/Ctr Nagasaki/Cps	-	LC512737 LC512738	Y Y
	Iflaviridae	Isahaya Culex Iflavirus (ICIFV)	Wuhan fly virus 4 (YP 009342337)	-	-	-	43	71	0.0	Nagasaki/Cps	-	LC513835	Y
	Iflaviridae	Yongsan Iflavirus 1 (YIFV1)	(AXV43887)	96	100	0.0	97	96	0.0	Tottori/Ctr	-	LC513837	N
	Iflaviridae	Yonago Culex Iflavirus (YCIFV)	Wuhan insect virus 13 (YP 009342321)	-	-	-	38	82	0.0	Tottori/Ctr	-	LC513836	Y
	*Sobemovirus*-related	Wenzhou sobemo-like virus 3 (WSLV3)	(APG75759)	91	92	0.0	96	47	0.0	Ishikawa/Ctr Tottori/Ctr Nagasaki/Ctr Nagasaki/Cps	-	LC512854 LC512855 LC512856 LC512857	N N N Y
	*Sobemovirus*-related	Bat sobemovirus (BSV)	(AGN73380)	90	42	0.0	94	42	4×10^−133^	Ishikawa/Ctr Tottori/Ctr Nagasaki/Ctr Nagasaki/Cps	-	LC512858 LC512859 LC512860 LC512861	N N Y Y
(−)ssRNA	Xinmoviridae	*Culex tritaeniorhynchus* anphevirus (CtAV)	Guadeloupe mosquito mononega-like virus (QEM39171.1)	-	-	-	38	47	0.0	Ishikawa/Ctr Tottori/Ctr	-	LC514054 LC514055	Y Y
	Rhabdoviridae	*Culex tritaeniorhynchus* rhabdovirus (CTRV)	(BAU46576)	99	100	0.0	99	56	0.0	Japan/Ctr	-	LC514403	Y
	Rhabdoviridae	*Culex pseudovishnui* rhabdo-like virus (CpRLV)	Tongilchon virus 1 (YP_009182186)	76	55	0.0	88	54	0.0	Nagasaki/Cps Nagasaki/Cps	-	LC514056 LC514057	Y Y
	Bunyavirales	*Culex pseudovishnui* bunya-like virus (CpBLV)	Bunyaviridae environmental sample (AJT39594)	80	98	0.0	-	-	-	Nagasaki/Cps Nagasaki/Cps	L	LC514291 LC514293	N Y
	Aspiviridae-related	*Culex tritaeniorhynchus* Aspi-like virus (CtALV)	*Fusarium poae* negative-stranded virus (YP_009272911)	-	-	-	30	80	0.0	Tottori/Ctr	-	LC514058	Y

Novel viruses are highlighted in blue. * Only the RNA-dependent RNA polymerase (RdRp) region’s identity is shown, except for bat sobemovirus. ^†^ Location is one of the following: Ishikawa, Tottori, Nagasaki, or Japan (when sequences obtained from different locations were identical). ^‡^ Species origin is abbreviations of Ctr (*C. tritaeniorhynchus*), Cps *(C. pseudovishnui*), and Cnt (*C. inatomii*). ^¶^ complete CDs: Yes (Y) and No (N).

**Table 3:** Viral diversity based on mosquito species and collection sites.

Virus Name	Mosquito Groups *				
	Ctr-Ishikawa	Ctr-Tottori	Ctr-Nagasaki	Cps-Nagasaki	Cnt-Tottori
WSLV3	+	+	+	+	−
BSV	+	+	+	+	−
CvsTV	+	+	+	+	−
HPLV22	+	+	+	−	−
CtPV	+	+	+	−	−
CtNLV	+	+	+	−	−
CtAV	+	+	+	−	−
CtFLV	+	+	+	−	−
HCLV1	+	+	−	−	−
CTRV	+	+	−	−	−
HMV2	+	+	−	−	−
HMV4	−	+	−	−	+
ICIFV	−	−	+	+	−
WTLV11	−	−	+	+	−
MFV	−	−	+	+	−
CxFV	−	−	+	−	−
JEV	+	−	−	−	−
YCIFV	−	+	−	−	−
CtALV	−	+	−	−	−
YIFV	−	+	−	−	−
CpPV	−	−	−	+	−
CpNLV	−	−	−	+	−
CpBLV	−	−	−	+	−
CpRLV	−	−	−	+	−
CpTLV	−	−	−	+	−
CiTV	−	−	−	−	+
CiLLV	−	−	−	−	+

* mosquito group abbreviations are Ctr (*C. tritaeniorhynchus*), Cps *(C. pseudovishnui*), and Cnt (*C. inatomii*).

## Supplementary Materials

The following are available online at https://www.mdpi.com/1999-4915/12/3/264/s1, Figure S1: (A) Phylogenetic analysis of Luteo-sobemo cluster using nucleotide sequences of segment 1. (B) Phylogenetic analysis of Luteo-sobemo cluster using nucleotide sequences of segment 2. Figure S2: (A) Phylogenetic analysis of Bunyavirus-like cluster using nucleotide sequences of M segment. (B) Phylogenetic analysis of Bunyavirus-like cluster using nucleotide sequences of S segment. Figure S3: (A) Seasonal changes in virome composition of *C. tritaeniorhynchus* mosquitoes in Tottori in 2017. (B) Seasonal changes in virome composition of *C. tritaeniorhynchus* mosquitoes in Ishikawa in 2017. Figure S4: Virome composition based on viruses’ presumptive host. Table S1: Percentage of viral reads from NGS. Table S2: Primers used in the study. Table S3: Virome identification result (additional viral segments). Table S4: Individual infection rate of several viruses.
