# Supplementary material for: Deciphering the Virome of Culex vishnui Subgroup Mosquitoes, the Major Vectors of Japanese Encephalitis, in Japan
Source: Viruses. 2020 Feb 28;12(3):264. doi: 10.3390/v12030264 (PMC7150981; doi:10.3390/v12030264)
Supplement: Supplementary file 1 [file viruses-12-00264-s001.zip › Supplementary Figures.pdf]

Supplementary Figures

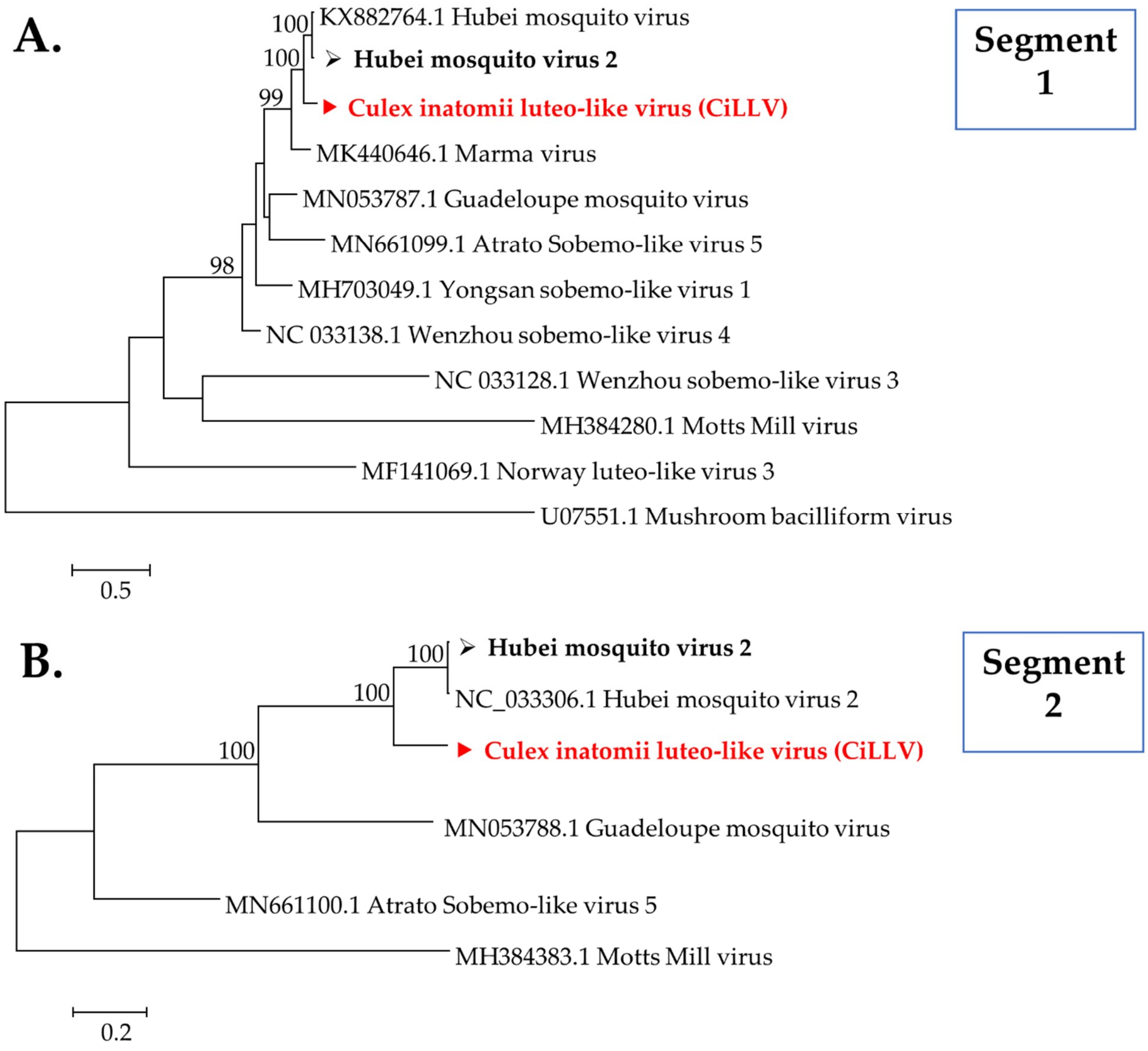

Figure S1. (A) Phylogenetic analysis of Luteo-sobemo cluster using whole nucleotide sequences of segment 1. **(B)** Phylogenetic analysis of Luteo-sobemo cluster using whole nucleotide sequences of segment 2. Analysis were performed with the maximum likelihood method and node bootstraps were calculated with 1,000 replicates. Bootstrap values < 70 are not shown. Viruses identified in this study are in bold, with ► indicating novel viruses and ➢ indicating a new strain identified in this study.

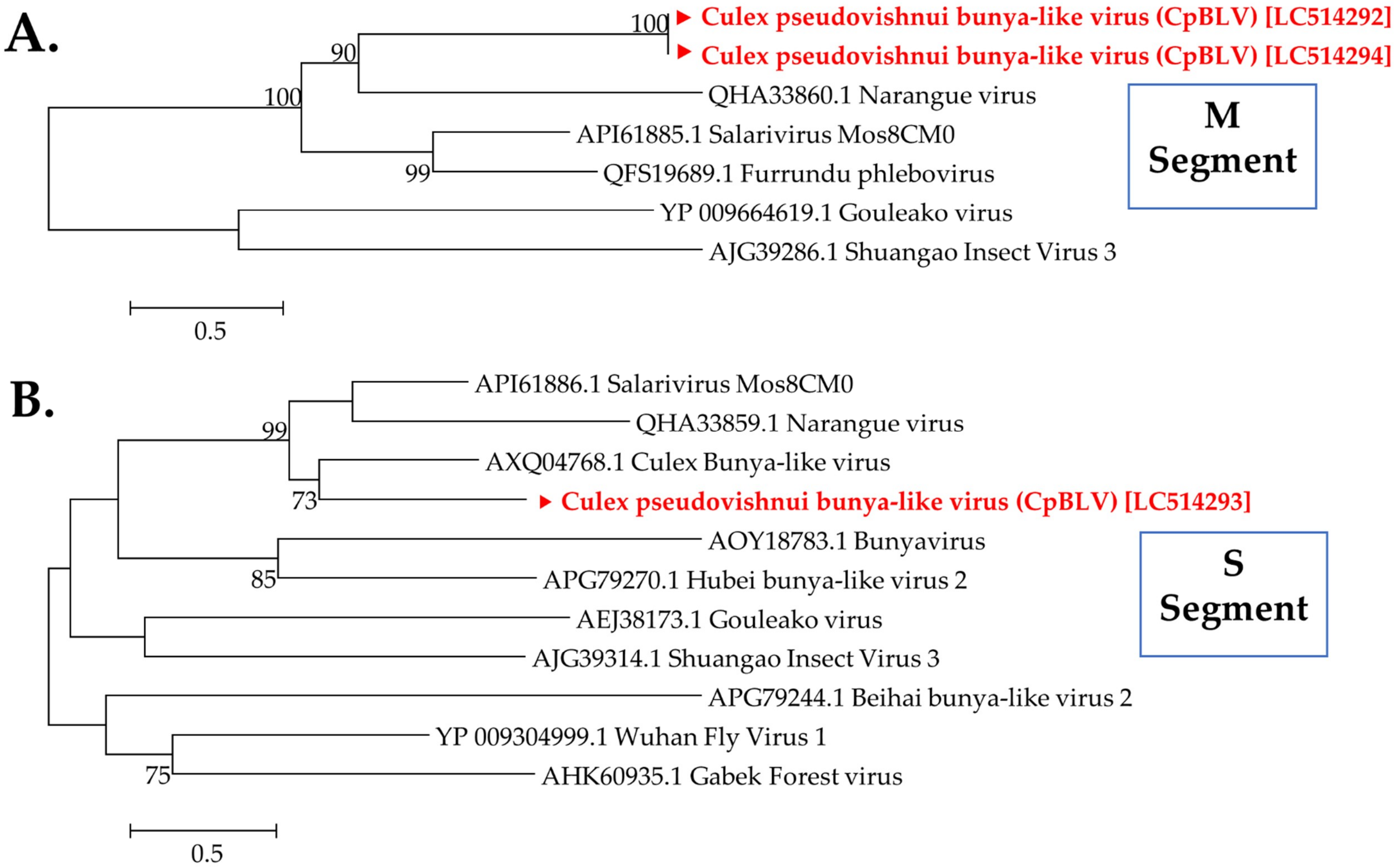

Figure S2. (A) Phylogenetic analysis of Bunyavirus-like cluster using whole nucleotide sequences of M segment. **(B)** Phylogenetic analysis of Bunyavirus-like cluster using whole nucleotide sequences of S segment. Analysis were performed with the maximum likelihood method and node bootstraps were calculated with 1,000 replicates. Bootstrap values < 70 are not shown. Viruses identified in this study are in bold, with ► indicating novel viruses and ➢ indicating a new strain identified in this study.

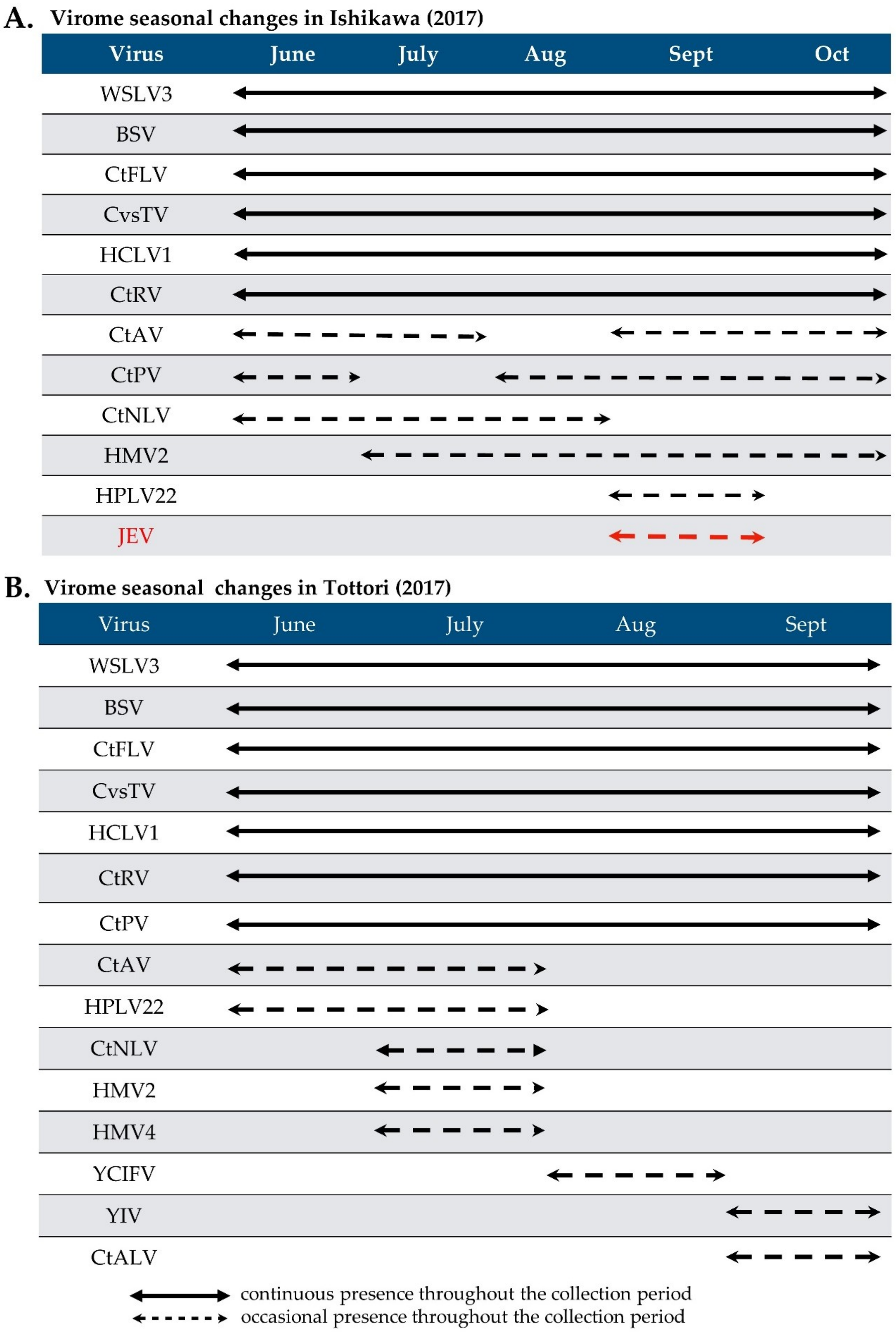

Figure S3. (A) Seasonal changes in virome composition of *C. tritaeniorhynchus* mosquitoes in Tottori in 2017. **(B)** Seasonal changes in virome composition of *C. tritaeniorhynchus* mosquitoes in Ishikawa in 2017.

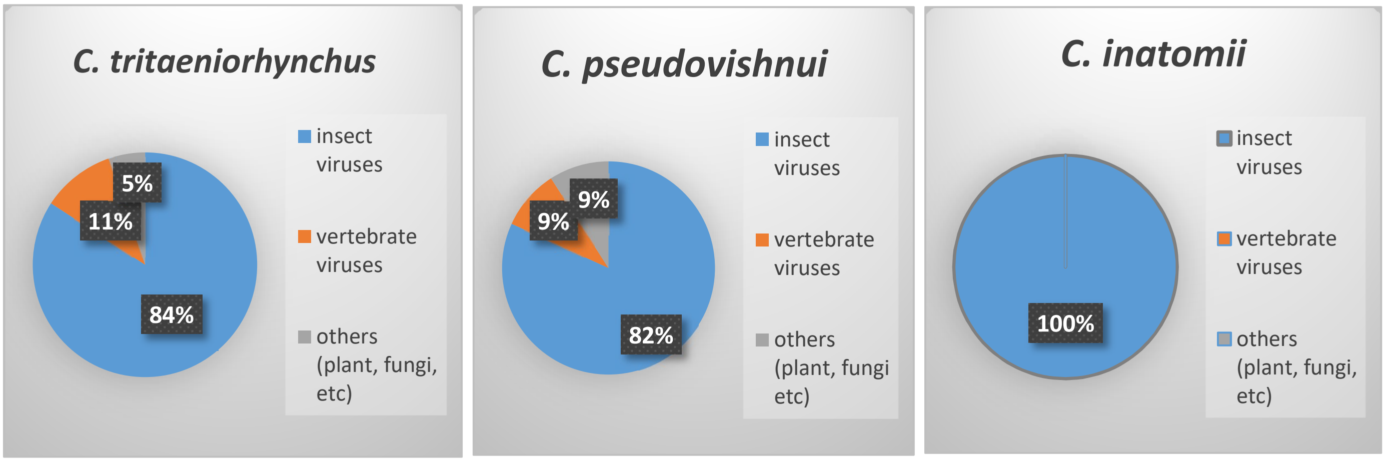

(A) (B) (C)

Figure S4. Virome composition based on virus host type. Insect viruses constituted most of viral genomes identified, as minimum as 84% in *C. tritaeniorhynchus* (A), 82% in *C. pseudovishnui* (B), or at most 100% in *C. inatomii* (C). Host type determination was based on previously reported studies and/or phylogenetic correlation (being in the same cluster) with insect, vertebrate, or other viruses (such as plant and fungi).
