# Supplementary material for: Deciphering the Virome of Culex vishnui Subgroup Mosquitoes, the Major Vectors of Japanese Encephalitis, in Japan
Source: Viruses. 2020 Feb 28;12(3):264. doi: 10.3390/v12030264 (PMC7150981; doi:10.3390/v12030264)
Supplement: Supplementary file 1 [file viruses-12-00264-s001.zip › Supplementary Table S1.pdf]

Table S1. Percentage of Viral Reads from NGS

Viruses	Percentage of Viral Reads (Virus Abundance) from NGS*																		
	1st NGS													2nd NGS*					
	Ctr-I 1	Ctr-I 2	Ctr-I 3	Ctr- I4	Ctr-I 5	Ctr-T 1	Ctr-T 2	Ctr-T 3	Ctr-N 1	Ctr-N 2	Cps-N 1	Cps-N 2	Cnt-T	Ctr 1	Ctr 2	Ctr 3	Ctr 4	Ctr-Cps 5	Cps 6
**dsRNA viruses**																			
CvsTV	0.010	0.007	0.059	0.043	0.008	0.044	0.052	0	0.018	0.047	0	0.026	0	0	0	0	0	0	0
CiTV	0	0	0	0	0	0	0	0	0	0	0	0	0.152	0	0	0	0	0	0
CpPV	0	0	0	0	0	0	0	0	0	0	0	0.005	0	0	0	0	0	0	0
HPLV22	0	0	0	0.103	0	0.047	0.422	0	0.866	0.022	0	0	0	0	0	0	0	0	0
CtPV seg1	0.116	0	0.312	0.833	0.216	0.270	1.054	0.445	0.192	0.859	0	0	0	0	0	0	0	0	0
CtPV seg2	0.288	0	0.829	2.696	0.971	0.979	3.135	1.472	0.616	2.951	0	0	0	0	0	0	0	0	0
HCLV1 seg1	0	0.020	0.022	0.027	0.033	0.009	0.025	0	0	0	0	0	0	0	0	0	0	0	0
HCLV1 seg4	0.004	0.012	0.023	0.018	0.020	0.008	0.007	0	0	0	0	0	0	0	0	0	0	0	0
**(+)ssRNA viruses**																			
CtFLV	0.056	0.035	0.021	0.098	0.041	0.0474	0.157	0.054	0.023	0.0602	0	0	0	0	0	0	0	0	0
JEV	0	0	0	0.078	0	0	0	0	0	0	0	0	0	0	0	0	0	0	0
CtNLV	0.038	0.159	0.342	0.052	0.134	0.154	0.530	1.407	0	1.017	0	0	0	0	0	0	0	0	0
CpNLV	0	0	0	0	0	0	0	0	0	0	0	1.182	0	0	0	0	0	0	0
CpTLV	0	0	0	0	0	0	0	0	0	0	0	0.017	0	0	0	0	0	0	0
HMV2 seg1	0	0.103	0.995	0.149	0.183	0.460	0	0	0	0	0	0	0	0	0	0	0	0	0
HMV2 seg2	0	0.049	0.493	0.076	0.093	0.223	0	0	0	0	0	0	0	0	0	0	0	0	0
CiLLV seg1	0	0	0	0	0	0	0	0	0	0	0	0	3.854	0	0	0	0	0	0
CiLLV seg2	0	0	0	0	0	0	0	0	0	0	0	0	1.416	0	0	0	0	0	0
HMV4	0	0	0	0	0	0.080	7.065	0	0	0	0	0	88.61	0	0	0	0	0	0
WTLV11	0	0	0	0	0	0	0	0	0.505	1.2162	0.357	1.118	0	0	0	0	0	0	0
YCIFV	0	0	0	0	0	0	0	0.466	0	0	0	0	0	0.043	0	0	0	0	0
YIFV	0	0	0	0	0	0.038	0	0	0	0	0	0	0	0	0	0	0	0	0
ICIFV	0	0	0	0	0	0	0	0	0	0	0.118	0.185	0	0	0	0	0	0	0
WSLV3	0.809	1.132	2.088	1.589	1.549	4.614	4.560	1.806	4.262	7.491	0.191	2.034	0	0	0	0	0	0	0
BSV	0.330	0.668	0.925	0.744	0.617	2.547	2.520	0.644	2.223	4.913	0	0.867	0	0	0	0	0	0	0

Percentage of Viral Reads (Virus Abundance) from NGS*

Viruses	1st NGS libraries													2nd NGS libraries**					
	Ctr-I 1	Ctr-I 2	Ctr-I 3	Ctr-I 4	Ctr-I 5	Ctr-T 1	Ctr-T 2	Ctr-T 3	Ctr-N 1	Ctr-N 2	Cps-N 1	Cps-N 2	Cnt-T	Ctr1	Ctr2	Ctr3	Ctr4	Ctr-Cps5	Cps6
**(-)ssRNA viruses**																			
CtAV	0.183	0.114	0	0.143	0.275	0.429	0.463	0.410	0.409	0.754	0	0	0	0	0	0	0	0	0
CTRV	0	0.545	1.433	1.421	1.164	0.667	0.467	0.330	0	0	0	0	0	0	0	0	0	0	0
CpRLV	0	0	0	0	0	0	0	0	0	0	0.638	0.637	0	0	0	0	0	0	0
CpBLV Lseg	0	0	0	0	0	0	0	0	0	0	0.199	0.426	0	0	0	0	0	0	0
CpBLV Mseg	0	0	0	0	0	0	0	0	0	0	0.047	0.169	0	0	0	0	0	0	0
CpBLV Sseg	0	0	0	0	0	0	0	0	0	0	0.312	0.22	0	0	0	0	0	0	0
CtALV	0	0	0	0	0	0.05	0	0	0	0	0	0	0	0	0	0	0	0	0
Total viral reads (%)	1.832	2.843	7.543	8.071	5.305	10.668	20.456	7.035	9.113	19.33	1.911	7.01	94.036	0.043	2.191	1.718	0	0	0
Total viral reads	21869	32672	129092	92640	59661	115751	245153	67550	29402	59761	7988	404705	222720	68	1462	888	0	0	0
Total raw reads	1.193.722	1.149.162	1.711.456	1.147.850	1.124.596	1.085.052	1.198.428	960.250	322.648	309.156	418.024	5.773.132	236.846	157.520	66.732	51.700	57.324	33.000	51.578

* Abbreviations are Ctr-I (*C. tritaeniorhynchus* from Ishikawa Pref.), Ctr-T (*C. tritaeniorhynchus* from Tottori Pref.), Ctr-N (*C. tritaeniorhynchus* from Nagasaki Pref.), Cps-N (*C. pseudovishnui* from Nagasaki Pref.), and Cnt-T (*C. inatomii* from Tottori Pref).

**Samples used were stored supernatants
