# Supplementary material for: Deciphering the Virome of Culex vishnui Subgroup Mosquitoes, the Major Vectors of Japanese Encephalitis, in Japan
Source: Viruses. 2020 Feb 28;12(3):264. doi: 10.3390/v12030264 (PMC7150981; doi:10.3390/v12030264)
Supplement: Supplementary file 1 [file viruses-12-00264-s001.zip › Supplementary Table S2.pdf]

Table S2. Primers used in the study

Primer name	Direction	Sequence
CiTV	Fw	ATGAGGTGGTCGACTGGTGG
	Rv	GGTCGAAAGAAGAAGAGTCGATGC
CtPV (RdRp) Seg1	Fw	TCCGGCAGTATGTGGACAAAC
	Rv	GTGGATACAAGAAGGAGGCAACT
CtPV Seg2	Fw	TTCCCACAATGCCTACGAATG
	Rv	TTTCCGTTCGGTATCGCATTG
HCLV1	Fw	CGCTT CTGGA GTTAA GTTGA GG
	Rv	GAGTT GTGCC TCAAC CAGG
HPLV22	Fw	GGTGT CACGT GAATG GAGAG C
	Rv	GTTCA CTTTG CCAAG AACCG C
CvsTV	Fw	TATCACTACTGCTGCCTCGACG
	Rv	CTCAAACTTCTCACTAGCAGTTGCC
CpPV	Fw	ATTCTTGCGGTGAGGAGTCG
	Rv	TATGGTGGCGCGATATGC
BSV	Fw	GCTCC ATACG TATCA GGCGT ACG
	Rv	GACGC TGGCA AATTC ATGGA CAG
HMV2	Fw	CTGTG ACCCT GATCG AGTCT C
	Rv	GTCCC AAGCT GATCT GTCTA G
CtNLV	Fw	TATGATGGATAGAGTTGAGCACG
	Rv	TAGCTATCTGCACAAGGGTATTC
CtFLV	Fw	ACTTGGCATGGTAAGGAAGG
	Rv	ATACCCAGAATTTGTATGTGCGG
YIFV	Fw	ATGACTAATGCCTTCACGTATGG
	Rv	ACTAGTCTCTGATTGTTTGTCGC
YCIFV	Fw	AATATAGCCGTTCATCCCCACCG
	Rv	GTCTACTTCATCCCACGACGG
ICIFV	Fw	TTCGAGGAGTGGACTATTCG
	Rv	TAACTTACCAACCTCCGTATAGC
CiLLV Seg1	Fw	GCTTCATTACATAGCGAATGACAG
	Rv	CCAGTACGCTGGATAACACG
CiLLV Seg2	Fw	TGAGCCGAAAGTACTACAGC
	Rv	GAGGTTCTTACCGAGCCACG
CpTLV	Fw	ACTGCTGCCGGATCTCAAGG
	Rv	CGTCATGCGATGGTACCTGTTG

CpNLV	Fw	AAAGGAGGCGGGATATTGG
	Rv	AGAACGGTTCTTCCTCGAATC
WTLV11	Fw	TTGATTGCCACGTCAACAGG
	Rv	GTTGTGCTTAATGGCTAGTTCC
WSLV3	Fw	GGCAGATTCCGTCTAATTTCC
	Rv	TCATGTCATACTGAACGGTCC
HMV4	Fw	CTACCTACCTACACCCGCTTG
	Rv	CCCAGTGTTTTGATCGCCTG
WTLV11	Fw	TTGATTGCCACGTCAACAGG
	Rv	GTTGTGCTTAATGGCTAGTTCC
HMV4	Fw	CTACCTACCTACACCCGCTTG
	Rv	CCCAGTGTTTTGATCGCCTG
CtFLV scaffold 1	Fw	GACTCACAGATAACACCAAAAG
	Rv	CCATTACCTCTTCTGTAGTCAC
CtFLV scaffold 2	Fw	CAACA GATGG AGGCA AATAC
	Rv	GTTAT CTGTG AGTCC TAAAA TGTC
JEV NS5	Fw	GCYGA RCAGA AYCAA TGGAG C
	Rv	GCTCC WAGCC ACATG AACCA
FU1 & cFD3	Fw	TACAACATGATGGGAAAGAGAGAGAA
	Rv	AGCATGTCTTCCGTGGTCATCCA
CTRV	Fw	CGGTT ACCGG GCTAT TAAAC G
	Rv	GAATG CGCCA TAAAC GGTTA TG
CtAV	Fw	CAACCAGTACTCCCAGCATGAGC
	Rv	TTTGTGAACGCTCTTCCGCTCTC
CtALV	Fw	TAACAGTCAAACTGGTTCTGACTCC
	Rv	CTCATTGCCTGTGAAAGAAGCATC
CpBLV Lseg	Fw	GGTGCTCAAAGCTTTAGCTGAC
	Rv	TGCAAAGTGTCCGAGAGTAG
CpBLV Mseg	Fw	GGTCTTCCAGGCAAACTAACTTG
	Rv	AGTGAATCTGCGAGCAGAGGAG
CpBLV Sseg	Fw	TGACTCTGGCTAGAATTGCAGC
	Rv	TCCAATAAGCTGATTCAGCCTAAG
CpRLV	Fw	AAGAAATTGATGCTCAGGGACTAC
	Rv	GAGACTTCTCAAAGAATTCGTG
