# Supplementary material for: Deciphering the Virome of Culex vishnui Subgroup Mosquitoes, the Major Vectors of Japanese Encephalitis, in Japan
Source: Viruses. 2020 Feb 28;12(3):264. doi: 10.3390/v12030264 (PMC7150981; doi:10.3390/v12030264)
Supplement: Supplementary file 1 [file viruses-12-00264-s001.zip › Supplementary Table S3-4.pdf]

Supplementary Tables

Table S3. Virome identification result (additional viral segments)

Virus category	Order, Family Genus	Virus name	Closely related viruses	Location* / species origin†	Segment	Accession no.	CDs
dsRNA	*Partitiviridae*	Culex tritaeniorhynchus partitivirus (CtPV)	Galbut virus (AWY11073)	Japan/Ctr	2	LC516822	Complete
	Chrysoviridae	Hubei chryso-like virus 1 (HCLV1)	(MF176283)	Tottori/Ctr	4	LC514397	Complete
(+)ssRNA	*Luteoviridae*-related	Hubei mosquito virus 2 (HMV2)	(KX882765)	Ishikawa/Ctr<br>Tottori/Ctr	2<br>2	LC513831<br>LC513832	Incomplete<br>Incomplete
	Luteoviridae-related	Culex inatomii luteo-like virus (CiLLV)	Hubei mosquito virus 2 (KX882765)	Tottori/Cnt	2	LC513834	Complete
(-)ssRNA	*Bunyavirales*	Culex pseudovishnui bunya-like virus (CpBLV)	Narangue virus (hypothetical protein) (QHA33860)	Nagasaki/Cps	M<br>M	LC514292<br>LC514294	Complete<br>Complete
	Bunyavirales	Culex pseudovishnui bunya-like virus (CpBLV)	Salarivirus and Culex Bunya-like virus (hypothetical protein) (MH188002)	Nagasaki/Cps	S	LC516823	Complete

Novel viruses are highlighted in blue.
*Location is one of the following: Ishikawa, Tottori, Nagasaki, or Japan (when sequences obtained from different locations were identical)
†species origin's abbreviations: Ctr, *Culex tritaeniorhynchus*; Cps, *Culex pseudovishnui*; Cnt, *Culex inatomii*.

Table S4. Individual infection rate of several viruses.

		No. of positive mosquitoes/total (%) *				
Collection Month	Sample	Culex tritaeniorhynchus anphevirus (CtAV)	Culex tritaeniorhynchus negev-like virus (CtNLV)	Culex tritaeniorhynchus flavi-like virus (CtFLV)	Culex vishnui subgroup totivirus (CvsTV)	Yonago Culex iflavirus (YCIFV)
Sept 2017	Misaki-machi Wajima-city	6/24 (25%)	ND**	2/24 (8%)	13/24 (54%)	ND
Sept 2017	Noto-machi Wajima-city	4/24 (17%)	ND	4/24 (17%)	14/24 (58%)	ND
June 2017	Monzen-machi Wajima-city	1/24 (4%)	0/24 (0%)	3/24 (12.5%)	12/24 (50%)	ND
Sept 2017	Monzen-machi Wajima-city	3/24 (12.5%)	ND	3/24 (12.5%)	8/24 (33%)	ND
July 2017	Yonago waterbirds sanctuary, Yonago city	3/24 (12.5%)	1/24 (4%)	8/24 (33%)	14/24 (58%)	ND
Aug 2017	Yonago waterbirds sanctuary, Yonago city	1/24 (4%)	ND	1/24 (4%)	12/24 (50%)	0/24 (0%)
Infection rate ranges		4-25%	0-4%	4-33%	33-58%	0%

*per total of 24 individual mosquitoes

**ND=Not Done
